# End-tidal CO_2_ response to passive leg raise for fluid management in lung resections: A randomized controlled trial

**DOI:** 10.12669/pjms.41.12.12679

**Published:** 2025-12

**Authors:** Havva Suheyla Akin Uzan, Lale Yuceyar, Nevzat Cem Sayilgan

**Affiliations:** 1 Havva Suheyla Akin Uzan, Department of Anesthesiology and Reanimation, Edirne State Hospital, Saglik Mah. Dr. Sadık Ahmet Cad. No: 8, 22030, Edirne, Turkiye. Department of Anesthesiology and Reanimation, Cerrahpaşa Medical Faculty, Istanbul University-Cerrahpaşa, Fatih, 34098, Istanbul, Turkiye; 2 Lale Yuceyar, Department of Anesthesiology and Reanimation, Department of Anesthesiology and Reanimation, Cerrahpaşa Medical Faculty, Istanbul University-Cerrahpaşa, Fatih, 34098, Istanbul, Turkiye. Cerrahpaşa Medical Faculty, Istanbul University-Cerrahpaşa, Fatih, 34098, Istanbul, Turkiye; 3 Nevzat Cem Sayilgan, Department of Anesthesiology and Reanimation, Department of Anesthesiology and Reanimation, Cerrahpaşa Medical Faculty, Istanbul University-Cerrahpaşa, Fatih, 34098, Istanbul, Turkiye, Cerrahpaşa Medical Faculty, Istanbul University-Cerrahpaşa, Fatih, 34098, Istanbul, Turkiye

**Keywords:** End-tidal CO_2_, Fluid responsiveness, Goal-directed therapy, Lung resection, Passive leg raise

## Abstract

**Objective:**

To evaluate whether changes in end-tidal carbon dioxide (EtCO_2_) following passive leg raise (PLR) can predict fluid responsiveness and guide fluid therapy in patients undergoing Lung Resection Surgery (LRS).

**Methodology:**

This prospective randomized controlled trial was conducted at Istanbul University-Cerrahpasa Medical Faculty Hospital, Istanbul, Turkey, between August 2020 and March 2021. Fifty patients undergoing elective LRS were enrolled. After anesthesia induction, EtCO_2_ was measured before and one minute after PLR. A ≥2 mmHg increase was considered responsive. Responders were randomized into a study group (fluid bolus) and a control group (maintenance fluid only); non-responders formed a third group. Hemodynamic parameters, fluid balance, urea, creatinine and lactate levels were recorded perioperatively.

**Results:**

The proportion of fluid responders was 62%. The study group received significantly more intravenous fluid and showed no cases of acute kidney injury (AKI), while AKI was observed in the control and unresponsive groups. Postoperative urea levels increased significantly only in the control group. Lactate levels rose intraoperatively in all groups but normalized within 24 hours. A positive correlation was found between surgical duration and lactate levels. EtCO_2_ and heart rate did not differ significantly between groups.

**Conclusion:**

EtCO_2_ changes in response to PLR may provide a simple, non-invasive indicator of fluid responsiveness in thoracic surgery. Targeted fluid supplementation in responsive patients appears to improve renal outcomes. Further studies are needed to validate these findings.

Registration No. ClinicalTrials.gov (Identifier: NCT06855966).

## INTRODUCTION

Lung resection surgeries carry a significant risk, with mortality rates ranging from 1% to 7%, depending on the extent of resection, surgical duration and patient profile.^1^,^2^ One-lung ventilation, direct pulmonary manipulation and ischemia-reperfusion injury increase the risk of pulmonary edema, particularly with excessive perioperative fluid administration.^3^ Conversely, overly restrictive regimens may lead to hypoperfusion and acute kidney injury (AKI). Therefore, achieving an optimal fluid balance is crucial in thoracic surgery.^4^ As a result, perioperative restrictive fluid management guided by dynamic circulatory parameters has become a key component of the enhanced recovery after surgery (ERAS) protocols.^5^ Despite recommendations against excessive preoperative fasting, hypovolemia is still commonly observed due to prolonged fasting, diuretic use, or age-related physiological changes.^6^ Accurate assessment of volume status is therefore essential.

Passive leg raise (PLR) is a dynamic, reversible and non-invasive maneuver used to assess fluid responsiveness in order to achieve a goal directed fluid therapy, by temporarily increasing cardiac preload.^7^ While methods like pulse contour analysis, esophageal Doppler and echocardiography are validated for preload assessment, their use is limited due to invasiveness, cost, or need for expertise. In contrast, capnography and end tidal carbon dioxide (EtCO_2_) measurement is a reliable and noninvasive entity to evaluate the PLR response. EtCO_2_ reflects pulmonary blood flow and can be used as an indirect marker of cardiac output under stable ventilation and metabolism. A ≥2 mmHg increases in EtCO_2_ following PLR has been shown to predict a >15% rise in cardiac output, correlating well with fluid responsiveness in ICU settings.^8^

Thoracic surgery presents unique monitoring challenges-such as open chest conditions and fluctuating intrathoracic pressures-which often render continuous cardiac output monitoring unavailable or unreliable in this setting.^9^ Thus, predicting the fluid deficit before thoracotomy seems more appliable. This study aimed to evaluate whether EtCO_2_ changes in response to PLR can predict fluid responsiveness and help optimize fluid management in thoracic surgery. Secondary objectives included assessing the impact of post-induction fluid correction on postoperative renal and metabolic outcomes and the utility of Pleth Variability Index (PVI) as a supplementary parameter.

## METHODOLOGY

This randomized controlled study was conducted at Istanbul University-Cerrahpasa Medical Faculty Hospital, between August 2020 and March 2021. The trial was retrospectively registered at ClinicalTrials.gov (Identifier: NCT06855966).

### Ethical Approval:

It was obtained from the Ethical Committee of Istanbul University-Cerrahpasa Medical Faculty, with document number 72109855-604.01.01-77663; dated: July 16, 2020. Written informed consent was obtained from all participants.

### Inclusion criteria:

Adult patients(≥18 years) scheduled for lung resection surgery (LRS) for lung resection.

### Exclusion criteria:

included ASA score >3, known cardiac or renal failure, need for postoperative ICU admission, and procedures beyond lobectomy.

Of 62 patients assessed, Fifty three met the inclusion criteria; three were excluded due to incomplete data, resulting in 50 patients included in the final analysis. Demographic characteristics, comorbidities, spirometric findings and exercise capacity were recorded preoperatively. Standard ASA monitoring included ECG, non-invasive blood pressure, pulse oximetry and end-tidal CO_2_ (EtCO_2_). Each patient was also monitored with Masimo Radical-7 for Pleth Variability Index (PVI). Anesthesia was induced with midazolam (0.05 mg/kg), fentanyl (2 mcg/kg), propofol (1–2 mg/kg) and rocuronium (1 mg/kg), followed by intubation with a double-lumen tube. Anesthesia was maintained with 0.5–1 MAC sevoflurane in volume-controlled ventilation (TV: 8 mL/kg, PEEP: 6 cm H_2_O). An arterial line was placed for invasive pressure monitoring and blood gas sampling.

After induction and stabilization, baseline EtCO_2_ was recorded. Passive leg raise (PLR) was performed by elevating the lower limbs 45° from the horizontal plane.^8^ A ≥2 mmHg increase in EtCO_2_ within one minute was defined as fluid responsiveness. Responsive patients were randomized by sealed envelope into two groups: a control group receiving maintenance fluid only (n=15) and a study group receiving an additional fluid bolus (n=16). Non-responders were allocated to the unresponsive group (n=19) and received standard fluid management. Following randomization, all patients were positioned in lateral decubitus and received regional analgesia with thoracic epidural catheter, paravertebral block, erector spinae or retrolaminar block on the operative side. Intraoperative fluid management per group is as following:

### Control group:

Received 2 mL/kg/h crystalloid. Hypotension (MAP <65 mmHg) was managed with vasopressors.

### Study group:

Received 250 mL crystalloid bolus at induction in addition to 2 mL/kg/h maintenance. An additional 250 mL bolus was given if MAP remained <65 mmHg. Vasopressors were used if hypotension persisted despite 500 mL fluid.

### Unresponsive group:

Managed with the same restrictive protocol as the control group. Vasopressors were used to maintain MAP ≥65 mmHg when needed.

Transfusion thresholds were 10 g/dL for patients with coronary artery disease and 8 g/dL otherwise. Intraoperative data included SAP, MAP, DAP, HR, SpO_2_, EtCO_2_ and PVI, recorded at six time points: pre-PLR, end of first minute of PLR, pre-incision, post-incision, end of surgery and pre-extubation. Additional readings were taken post-fluid bolus in the study group. Arterial blood samples were obtained at four time points: post-intubation, pre-extubation, arrival in the recovery unit and 24 hours postoperatively. Pre- and postoperative urea and creatinine levels were recorded. Perioperative fluid intake, urine output and fluid balance were calculated and monitored during the first 24 postoperative hours.

### Statistical analysis:

Statistical analysis was performed using MedCalc (v12.7.7). Data distribution was evaluated by Shapiro-Wilk test. Continuous variables were expressed as mean ± standard deviation (SD), median (min–max) and compared using Mann–Whitney U or Kruskal–Wallis test, as appropriate. Categorical variables were analyzed with chi-square or Fisher’s exact test. Repeated measures were analyzed with Wilcoxon or Friedman test. Correlations were assessed using Spearman’s rho. A p-value <0.05 was considered statistically significant.

## RESULTS

A total of 50 patients (37 males, 13 females) were analyzed. Demographic characteristics, including gender, weight, height, BMI, ASA scores, comorbidities, exercise capacity and spirometry results were comparable among the three groups. However, the study group had a significantly higher mean age (p=0.006). Surgical duration and laterality were similar across groups. Perioperative bleeding and vasopressor requirement did not differ significantly. Despite significantly higher fluid infusion rates and net fluid balance in the study group compared to the control and unresponsive groups (p=0.001 and p=0.006, respectively), urine output per kilogram per hour did not show a statistically significant difference between the groups (Table-I).

**Table-I:** Comparison of perioperative bleeding, urine output, fluid infusion rate and net fluid balance across groups.

Parameter	Study Group (n=16)	Control Group (n=15)	Unresponsive Group (n=19)	P^1^
Perioperative bleeding (ml)	126.67 ± 203.42	128.13 ± 141.38	144.74 ± 140.33	0.594
Vasopressor (total mcg)	0.05 ± 0.12	0.02 ± 0.06	0.07 ± 0.28	0.681
Urine output (ml/kg/hr)	1.14 ± 0.46	1.41 ± 1.04	1.46 ± 1.48	0.992
Fluid infusion rate (ml/kg/hr)	3.44 ± 1.34	2.27 ± 1.23	2.19 ± 0.69	0.001
Fluid balance (ml/kg/hr)	1.81 ± 1.30	0.51 ± 1.44	0.43 ± 1.58	0.006

*Data are presented as mean ± SD.*

1 *Kruskal-Wallis test.*

MAP values were significantly lower in the study group compared to the control and unresponsive groups before and during the PLR maneuver and again at the end of surgery (p < 0.05). Pre-extubation MAP was higher in the unresponsive group than in both other groups, while heart rate and EtCO_2_ showed no significant differences at any time point.

Baseline PVI values before PLR were significantly higher in the study group compared to the control and unresponsive groups (p = 0.02). Following PLR, this difference disappeared and was not observed in subsequent measurement periods. Unexpectedly, mean PVI increased after PLR-exceeding both the baseline value and the 13% cutoff previously suggested.^10^ However, no significant differences were found between the groups and PVI remained slightly above the reference threshold at all subsequent time points, including before incision, after incision, at the end of surgery and before extubation.

Regarding blood gas analyses, lactate levels increased significantly from baseline to the recovery unit in all groups (p<0.01 for each group). Although the control group showed the highest absolute increase, intergroup comparisons did not reach statistical significance. Within-group analysis demonstrated that recovery room and 24 hours postoperative lactate levels were both significantly higher than post-intubation levels across all groups. Surgical duration correlated positively with both pre-extubation (r = 0.533, p < 0.001) and recovery room lactate values (r = 0.438, p = 0.001), suggesting that longer procedures contributed to transient elevations in lactate (Table-II).

**Table-II:** Correlation Analysis of Lactate Levels and Surgery Duration at Different Time Points.

		Surgery Duration
Post intubation	r	0,022
	p	0,880
Pre extubation	r	0,533
	p	<0,001
Recovery	r	0,438
	p	0,001
Postoperative 24th hour	r	0,131
	p	0,366

Correlation with surgery duration was analyzed using Spearman’s rank correlation coefficient. Statistically significant values were considered at p < 0.05.

Preoperative and 24 hours postoperative urea levels were comparable between groups. However, only the control group exhibited a significantly higher postoperative urea levels than preoperative levels as shown at Table-III (p = 0.034). Creatinine levels did not differ significantly between or within groups at any time point.

**Table-III:** Comparison of preoperative and postoperative urea levels in all groups.

		Study	Control	Unresponsive	
		Mean.+SD	Mean.+SD	Mean.+SD	p^1^
Urea	Preoperative	36,13±11,78	29±12,64	35,15±22,54	0,189
	Postoperative	40,73±19,22	37,56±16,51	39,37±23,22	0,870
	p^2^	0,221	0,034	0,087	

Values are expressed as mean ± SD. Kruskal Wallist test^1^, Wilcoxon test^2^.

Regarding the 24 hours measurement, stage-I acute kidney injury (AKI) was identified in three patients from the control group and one patient from the unresponsive group. No cases occurred in the study group. At 48 hours, Stage-I AKI was identified in one patient from the control group and one from the unresponsive group.

## DISCUSSION

Our study supports the use of end-tidal carbon dioxide (EtCO_2_) changes in response to passive leg raise (PLR) as a non-invasive and easily accessible guide for fluid management in thoracic surgery. While EtCO_2_ response has predominantly been applied in patients with shock in the intensive care,^11^ our findings indicate that this parameter may also serve as a practical intraoperative tool to assess fluid responsiveness without requiring additional expertise or specialized equipment. Importantly, our work represents one of the few perioperative studies utilizing EtCO_2_ response to PLR as a surrogate of preload responsiveness.

Our finding that 62% of patients were fluid responsive is in line with studies employing EtCO_2_ as well as other dynamic PLR-based indices yet represents a slightly higher proportion than previously observed.^12^ We adopted a 2-mmHg increase in EtCO_2_ as the cut-off value; however, Raj et al.^12^ who regarded 1-mmHg as the threshold, also reported a relatively low specifity of EtCO_2_ in a response to mini-fluid challenge (MFC). Additionally, PLR has been shown to be more reliable than MFC for assessing fluid responsiveness.^13^,^14^ Thus, we believe that the difference in our study is more likely attributable to prolonged preoperative fasting rather than to a relatively limited specificity of EtCO_2_.

There were no statistically significant differences between groups in terms of concomitant diseases and demographic data, except for age. The lower baseline MAP in the study group may be explained by impaired autoregulatory mechanisms in elderly patients, particularly in response to anesthesia-induced hypovolemia and prolonged fasting.^15^,^16^ The study group received a fluid bolus after PLR, which appeared to correct this deficit, as the differences between groups disappeared before incision. These findings suggest that elderly or volume-depleted patients may benefit from targeted fluid supplementation early in the perioperative period.

All patients were monitored with the Masimo Rainbow SET® pulse co-oximeter (Masimo Corp., Irvine, CA, USA) for observational purposes only, due to the limited reliability of PVI in open-chest, one-lung ventilation cases.^17^ Monitoring was discontinued after thoracotomy. Baseline PVI, measured post-intubation but before PLR, was significantly higher in the study group, suggesting that the hemodynamic impact of hypovolemia may be more prominent in elderly patients.^18^,^19^ This difference disappeared post-PLR, yet PVI remained above the 13% threshold in all groups-contrary to expectations. It should also be noted that the threshold value considered differently in various studies.^20^ Since we did not conduct a subgroup analysis based on surgical type, potential differences in PVI reliability between thoracotomy and thoracoscopic procedures could not be evaluated.

Vasopressor use was similar across three groups, indicating that overall hemodynamic stability was maintained. However, the presence of fluid responsiveness in apparently stable patients highlights an important point: fluid responsiveness does not equate to fluid need.^21^ This concept has been emphasized in recent literature, advocating for a more tailored, goal-directed approach to volume therapy.^22^

Urine output remained below 2 mL/kg/h across all groups. This may reflect anesthesia-induced hormonal changes rather than true hypovolemia or renal dysfunction.^23^ Similar findings have been reported in thoracic surgery literature, suggesting that targeting urine output alone is not a reliable strategy for preventing AKI and may lead to fluid overloading.^24^

No significant differences were observed in pre- and postoperative creatinine levels between or within groups. However, according to KDIGO criteria, Stage I AKI occurred in three patients in control and one patient in unresponsive group at 24 hours. Due to early discharge under ERAS protocols, 48 hours AKI evaluation was not possible in 11 patients. Among the remaining 39, two developed Stage-I AKI (1 control, 1 unresponsive). It is noteworthy that the patients with Stage-I AKI at 48 hours were not the same individuals identified at 24 hours. All AKI cases occurred in the control and unresponsive groups, suggesting that restrictive fluid strategies may predispose to renal injury irrespective of fluid responsiveness. In a retrospective analysis of open thoracotomy patients, Kim et al. (2020)^24^ found no significant differences in AKI incidence across groups stratified by fluid volume, although a trend toward reduced AKI was observed with higher fluid administration. Notably, fluid therapy in that study was not individualized by goal-directed strategies, which may partly explain the absence of significant intergroup differences. Taken together, these findings highlight that adequate and tailored fluid administration may play a protective role against perioperative renal injury, although larger trials are needed to confirm this association. Because of the modest sample size, the AKI findings remain descriptive and cannot support firm conclusions. Nevertheless, the observed postoperative increase in urea levels compared with preoperative values may also suggest a degree of renal injury.

Lactate levels rose in all groups following surgery but normalized within 24 hours. Given the absence of sustained hypotension or high vasopressor requirements, this pattern likely reflects localized tissue hypoperfusion during surgical manipulation, together with the time-dependent relationship between surgical duration and lactate accumulation as emphasized by previous studies.^25^,^26^

Our results align with previous studies emphasizing the complexity of fluid management in thoracic surgery.^27^,^28^ Pulmonary protection must be balanced with the risk of hypoperfusion and simple parameters like urine output or MAP may not fully capture perfusion adequacy. While esophageal Doppler and pulmonary artery catheters provide more detailed hemodynamic data, their invasiveness, cost and technical complexity limit their widespread use; notably, they have not been shown to offer clear advantages over macrohemodynamic parameters in goal-directed fluid therapy.^29^ EtCO_2_, in contrast, is widely available, non-invasive and interpretable by most anesthesiologists.

### Limitations:

First, the relatively small sample size and single-center design of the study prevents definitive conclusions especially in terms of renal injury. Nevertheless, our sample size is consistent with other randomized controlled trials which conducted in thoracic surgical anesthesia focusing on fluid management.^30,31^ Second, we did not evaluate postoperative pulmonary complications, length of hospital stay, or other clinical outcomes, which would have provided further insight into the clinical significance of our findings. Third, the assumption that the PLR is the only factor influencing the cardiac output, while potential determinants such as metabolic rate were not considered. Fourth, the PLR may potentially decrease alveolar dead space, leading to an increase in EtCO_2_, which could be misinterpreted as an increase in CO. Latter two limitations could also explain the heterogeneity of the sensitivity and specificity of EtCO_2_ as a response to PLR.^32^

## CONCLUSION

Our findings suggest that EtCO_2_ monitoring may serve as a simple, non-invasive, and accessible tool to identify fluid-responsive patients under general anesthesia. By enabling early recognition of fluid responsiveness, PLR- and EtCO_2_-guided assessment has the potential to support more individualized and safer perioperative fluid management. Future larger, multicenter randomized trials and studies assessing outcomes such as postoperative pulmonary complications and length of hospital stay may be beneficial.

### Future recommendations:

In further investigation into EtCO_2_-guided fluid therapy in thoracic surgery. Integration of such dynamic and easily accessible tools into intraoperative practice may enhance individualized fluid management and improve postoperative outcomes.

### Authors’ Contribution:

**HSAU:** Conceptualization, data collection, manuscript drafting and revision, also responsible and accountable for the accuracy or integrity of the study and acts as the guarantor.

**LY:** Study supervision, critical review of the manuscript and final approval.

**NCS:** Methodological support, literature review and contribution to interpretation of results.

All authors have read and approved the final version of the manuscript.
